# The Greenland Glaciers

**Published:** 1882-05

**Authors:** C. H. Hitchcock


### THE GREENLAND GLACIERS.

The study of the Greenland glaciers takes us a step farther toward the understanding of the American ice-sheet. We have hardly appreciated the size of this island—it being larger than the Alpine glaciated tract. It is over twelve hundred miles long and four hundred broad, or as far as from Boston to the Missouri River. The interior is covered by a field of ice never crossed in a direct line by any civilized being. From three points attempts have been made to learn something of its nature. In 1830 Keilsen went eighty miles inland from Holsteinberg—latitude

67°—reaching the edge of the ice-sheet, which could not be climbed. In 1870 Nordenskjöld went in a distance of thirty miles, reaching the altitude of twenty-two hundred feet. He observed that the ice rose gradually toward the interior. The outer edge is a high, precipitous wall. Once entered upon the broad surface of the ice, it is like travelling upon the sea, away from all sight of land. From North Greenland, Dr. Hayes penetrated to a distance of seventy miles. It was a day's journey from the sea to the wall of ice. The second day was spent in climbing to the table-land ; the third day allowed a progress of thirty miles, the angle of ascent falling from six to two degrees. On the fourth day an altitude of five thousand feet was attained, and the ice still continued to rise, but because of inclement weather, no further progress was practicable. The view was that of a frozen Sahara, immeasurable to the human eye.

It is probable that Greenland slopes westerly in general, having its main axis of elevation near the eastern border. It may be compared to a broad platter inclined westerly, with occasional chinks in the sides through which the ice discharges itself as if it were a viscous body. The principal discharge of icebergs is upon the western side into Baffin's Bay. Not less than thirteen glaciers are found upon the western side to the south of Upernavik, about 73° north latitude, and the largest ones occur farther north. Some of the bergs are three thousand feet thick. The Humboldt glacier empties into Smith's Sound with a width of sixty miles, and showing ice-cliffs from fifty to three hundred feet high above the water. The rock-cliffs adjoining are from five hundred to one thousand feet high. At Polaris Bay a northward transportation is indicated, where Dr. Bessel found numerous granitic blocks containing peculiar garnets, such as abound in South Greenland, resting upon silurian limestones. Other glaciers have been mentioned farther north. Some have suggested that a series of islands will be found underneath the ice. Little is known of the eastern side, because it is practically inaccessible.—PROFESSOR C. H. HITCHCOCK, *in Popular Science Monthly for December.*

